# Secure and Robust Demand Response Using Stackelberg Game Model and Energy Blockchain

**DOI:** 10.3390/s23208352

**Published:** 2023-10-10

**Authors:** Mikhak Samadi, Sushmita Ruj, Henry Schriemer, Melike Erol-Kantarci

**Affiliations:** 1 School of Electrical Engineering and Computer Science, University of Ottawa, Ottawa, ON K1N 6N5, Canada; msama043@uottawa.ca (M.S.); hschriemer@uottawa.ca (H.S.); 2 School of Computer Science and Engineering, University of New South Wales, Sydney, NSW 2052, Australia; sushmita.ruj@unsw.edu.au

**Keywords:** energy blockchain, mixed-strategy Stackelberg game, consensus algorithm, smart contract, stochastic DR model

## Abstract

Demand response (DR) has been studied widely in the smart grid literature, however, there is still a significant gap in approaches that address security, privacy, and robustness of settlement processes simultaneously. The need for security and robustness emerges as a vital property, as Internet of Things (IoT) devices become part of the smart grid; in the form of smart meters, home energy management systems (HEMSs), intelligent transformers, and so on. In this paper, we use energy blockchain to secure energy transactions among customers and the utility. In addition, we formulate a mixed-strategy stochastic game model to address uncertainties in DR contributions of agents and achieve optimal demand response decisions. This model utilizes the processing hardware of customers for block mining, stores customer DR agreements as distributed ledgers, and offers a smart contract and consensus algorithm for energy transaction validation. We use a real dataset of residential demand profiles and photovoltaic (PV) generation to validate the performance of the proposed scheme. The results show the impact of electric vehicle (EV) discharging and customer demand reduction on increasing the probability of successful block mining and improving customer profits. Moreover, the results demonstrate the security and robustness of our consensus algorithm for detecting malicious activities.

## 1. Introduction

Energy within the smart grid context is an increasingly sophisticated problem that includes cyber security concerns, user privacy issues, and settlement processes. A useful framework to address this sophistication is provided by the energy IoT, where a consistent hierarchical architecture that connects sensing and actuating nodes can be implemented for the supervision of both loads and energy resources [1,2]. In modern grids, supervisory systems maintain the balance between generation and consumption using increasingly sophisticated mechanisms to incorporate greater economic flexibility and resource availability. Mechanisms such as demand response, household energy management [3,4,5], incentive-based DR offers [6], distributed energy resources (DERs) regulation [7], and energy trading markets [8] have been proposed and implemented. However, cyber security concerns, user privacy issues, potential node failures, uncertainties in the responses to demand control, and high processing times must be considered at the architectural level to ensure resilience and reliability within electricity and data exchange networks.

It is appealing to consider the role an energy blockchain can play in this regard. By operating an energy management system within a blockchain framework, this energy approach can diversify the potential responses of stakeholders into disparate domains while accommodating enhanced functionality. It allows energy to be monetized according to the activity undertaken within a collective response to its management. It recognizes that individual customers may have distinct energy consumption roles and responses to financial incentives that are not deterministically apparent to utilities seeking an aggregate demand reduction in their service area. We implement blockchain at the top of our incentive-based energy management system. Our proposed management system controls a utility company’s energy generation and cost using the distributed electricity generation and computational resources of the individual customers. The utility company via control agents asks customers, in a day-ahead system, to reduce their demand—which may include block mining—providing financial rewards as compensation for compliance. The blockchain is a distributed ledger to enable smart contracts in response to customer engagement with DR requests [9]. Customers, therefore, have two important roles, they can reduce their energy consumption to lower peak consumption, and they can collaborate in the block mining process using their computational and distributed energy resources.

With the increasing diversity of distributed energy resources (which include evolving load categories such as EV charging [10,11]), the high cost of supporting peak electricity consumption through centralized generation can be overcome at the distribution level if bottleneck issues associated with consumer participation in energy management can be addressed in the context of the likelihood of individual responses to distributed requests. The weakness of most DR approaches is an architectural one, a failure to accommodate the likelihood of the scope of individual responses to DR requests through a privacy-by-design probabilistic framework that is both fair and transparent, and that simultaneously expands and monetizes the energy informatics’ role of the individual consumer using a robust and secure energy blockchain whose consensus algorithm addresses reputation, DR availability, and compliance.

We propose a collaborative DR scheme and game theoretic approach to exploit the implicit stochastic nature of customer participation within a DER management context that is empowered by blockchain technology. This incentive-based DR scheme is expressed within a transactive model that negotiates with customers to find an optimal settlement point for customer energy reduction and reward price. Our model is based on control agents (CA) that act under the auspices of a distribution system operator (DSO). DR settlement is conducted via a private blockchain (PB), where the CA is a leader and customers are followers, for next-day demand reduction. This yields a distributed and stable data storage system that provides high cyber security, user privacy, and data accuracy while permitting customer use of the blockchain to monetize surplus energy generation as a reward for block mining.

Our approach distinguishes itself in two ways: (1) the use of a mixed-strategy Stackelberg game model that inherently accommodates the scope of potential customer response in a probabilistic fashion to achieve the best contributions that maximize customer reward; and (2) the use of a private blockchain to secure such contributions in a scalable fashion. Moreover, these two features are coupled. The mixed-strategy Stackelberg game is a stochastic model that considers all possible DR contributions (strategies) from players (customers and the CA) via their likelihood (probability values), where the tradeoff between DR response and the investment of surplus energy (i.e., excess DER generation) into block mining is explicitly considered. Specifically, this is a novel stochastic game model for customer energy consumption where the probability of being selected as a block miner depends on a customer’s history of energy consumption. This model provides customers with their best strategies to collectively monetize both DR and block mining. Furthermore, we study the robustness of this energy blockchain system against different security attacks by introducing a smart contract and consensus algorithm for transaction validity and authenticity.

The main contributions of this study can be summarized as follows:

- An incentive-based leader–follower stochastic DR model is proposed using blockchain to determine the random energy consumption of customers during peak hours, and effectively control the block mining. This model is a mixed-strategy Stackelberg game designed between the CA and customers, where customers are blockchain nodes and cooperate in block mining.
- For the mixed-strategy Stackelberg game, we prove the existence of the equilibrium point of the game based on the optimal solutions of customers and CA. The optimal solution is achieved by dividing the main Stackelberg game into multiple mixed-strategy subgames and finding a mixed equilibrium in each.
- The blockchain architecture in this work enables a secure, robust, and reliable distributed energy management system, while the processing and computational cost of the crypto algorithm is distributed across the network.
- We propose a novel consensus algorithm based on the proof of energy saving (PoES), where it selects a block miner considering the historical reputation, adequate energy resources (Availability), and DR contribution (Compliance ratio) among participants.
- The simulation results show that the proposed architecture is secure and robust against different cyber security attacks, and it is immune against 51% attack. We illustrate that the malicious nodes can be detected and penalized even in a small network with 10 active nodes.

The remainder of this paper is organized into seven sections. Section 2 presents a study on different existing energy blockchain architectures, Section 3 defines our proposed scheme, and Section 4 discusses the consensus model and smart contract algorithm. Section 5 presents the utility functions, strategies, and constraints for the CA and customers in addition to equilibrium analysis. Section 6 determines the data consistency and security of our proposed model. In Section 7, the simulation results are presented, and finally, Section 8 concludes the paper with a summary of our findings.

## 2. Literature Review

In this section, we review the dominant and recent literature on DR approaches with energy blockchain to place our work in context, and then differentiate it. We first begin with a brief overview of blockchain in this context. Blockchain can be simply defined as a ledger, which is securely distributed among the network nodes while its content cannot be altered [9]. Distributing computational cost in the blockchain is a unique feature suitable for many smart grid applications [12]. Initially, energy management has benefited from blockchain technology in data administration [7,13]. Later, blockchain became the point of interest in energy trading [14], energy storage [15], and security assurance [16]. Using the distributed ledger, smart contract, and consensus algorithm features of the blockchain can guarantee the performance of DR transactions in the smart grid. Distributed ledger architecture can work for DR agreements [17] between customers and utility companies, while the smart contract checks the transaction validity and user identity.

Blockchain is recommended for energy management systems because of its distributed architecture and secure functionality. This is illustrated in [18], where a data trust model and node reputation technique were represented in a private blockchain, with node trustworthiness calculated via reputation, confidence value, and observation. In Ref. [19], the functionality of private and public keys, and coin deposit for node registration and authentication, was noted. For a security enhancement solution, a two-stage security model with reputation-based voting and block miner selection dependent on the node history was developed [20]. In another work, a trust model was developed with reputation, endorsement, and confidence factors to evaluate transactions [21].

Smart contracts in the blockchain are mainly used to verify the validity of transactions and the sender’s identity [22]. A consensus algorithm specifies the rules of block miner selection and block generation for a blockchain network. For example, a proof of energy (PoE) consensus algorithm for energy trading between customers was presented in [14]. Virtual power plants (VPPs) control interactions and the VPP, with the minimum variation between consumption and production selected as a block miner. In a similar study, an energy trading model was given by a double auction between the customers and DERs [23]. Here, VPPs handle the tradings, and the one with the maximum-traded energy selects as a block miner based on the proof of energy market (PoEM) algorithm. Two consensus algorithms, proof of energy generation (PoEG), and proof of energy consumption (PoEC) have been described [24]. The former increases the energy production of prosumers, and the latter incentivizes prosumers to reduce consumption at peak times. To employ customers in the mining procedure, the authors in [25] presented a DR model where smart meters with adequate energy and processing hardware were selected as block miners.

Game-theoretic models have been used in blockchain to detect security attacks and to discover honest and selfish miners [26,27]. According to [28,29], game theory and advanced blockchain integration can guarantee profit maximization and secure transactions in microgrids. For instance, in [30], an extensive form game was presented to detect data collisions. Cheating actions among prosumers have also been determined using game models [16,31]. A game model can also be used for block miner selection. In Ref. [32], a non-cooperative game among agents to select a block miner based on its mining reward and computational resources was given.

Over the past few years, integrating demand response (DR) approaches with energy blockchain has been widely studied. The solutions have focused on interactive energy management systems [33]. Among the game theoretic approaches to DR management, Stackelberg games have become popular [34,35]. This is due to the hierarchical equivalence between game and management. In such games, one player (the leader) proposes its strategy, and then other players (followers) reply accordingly with their strategies [36]. This process repeats until all players reach their optimal outputs. For instance, a price-based DR model was presented in [37], where a Stackelberg game and blockchain were used to build a trading market between the prosumers. The model in [3] used blockchain with a distributed energy management system to optimize the energy consumption of household appliances to maximize the reward in response to DR signals. Article [38] has presented a blockchain-based collaborative power trading solution for 5G-enabled vehicular networks to enhance social welfare and reduce grid load. In a similar work, [39], authors have minimized electric vehicle charging costs and ensured the balance of power supply and demand while utilizing blockchain technology for transaction efficiency and privacy security. In Ref. [6], a DR model was implemented to determine the load shed availability of customers at peak times, in addition to using blockchain for transparent communication and a secure platform. Authors in [40] presented a blockchain-enabled peer-to-peer energy trading model that uses smart contracts and distributed ledger features to secure communications among prosumers and deal with cyberattacks. In a similar approach, a privacy and confidentiality guarantees model for prosumers during peer-to-peer energy trading is presented that utilizes public blockchain (Ethereum) and smart contracts [41].

Blockchain-based approaches can accommodate risk and demand uncertainty [42], but such functionality is not inherent to all game theoretic approaches, such as those that dominate DER management, as described above. Among such, there are no stochastic incentive-based demand reduction systems that use customers’ energy and potential resources for blockchain management to increase the stability and security of demand response data management. Unlike such energy blockchain approaches, our scheme implements a mixed-strategy Stackelberg game to probabilistically accommodate the breadth of customer energy consumption scenarios and to effectively control block mining. This is distinct from our prior deterministic approach [43]. In addition, we use smart contract and authentication schemes, such as a digital signature algorithm and public–private keys, for energy transactions’ authentication and validation to reduce cyber security attacks. Specifically, we develop a new consensus algorithm—proof of energy saving (PoES)—for the block mining process, where customer demand reduction affects its value directly, and the consensus value affects the customer’s utility function.

## 3. System Model

### 3.1. Network Model

**Figure 1:**
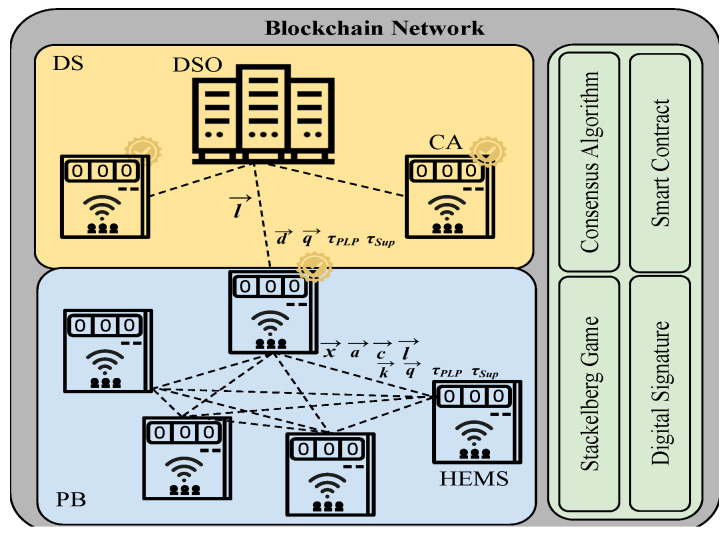
Hierarchical architecture and exchange parameters between DSO, certified CAs, and HEMSs.

As shown in Figure 1, the electricity grid can be divided into private blockchains comprised of multiple customers. To efficiently handle a large-scale demand response network, we divided the network into smaller networks as PBs to increase scalability. PB groups can be assigned considering geographical and regional situations or power flow constraints of the grid where customers perform as blockchain nodes. Note that while all parties to the local DR request overseen by the CA must be part of the PB, trust is explicitly not presumed. On the contrary, we consider that some nodes may act maliciously, and we employ the PB to detect such activity so as to ensure the security and robustness of our approach. The DSO monitors the energy transactions of the customers through multiple CAs. Each CA in its PB has a copy of blocks and regularly sends them to the DSO for data storage and monitoring purposes. In terms of technology, CA is similar to HEMS, but it is certified and trusted by the DSO. CAs are under the authority of DSO, and both are trusted parties in the network. According to Figure 1, data synchronization (DS) and PB networks are connected through wireless communication access points. At the DS, the CAs pass a copy of their local blocks to the DSO, and no CA knows the blocks belonging to their neighbors. The CAs are part of the DSO; they could be physical units installed in a local area or software as a service (SaaS) on the cloud. CA failure may happen due to a software or hardware disruption that could be resolved by repairing the unit or debugging the software and returning the CA to a fully functional service. In the interim, another CA (a CA from the neighborhood) takes action and controls the local area. Hence, the DSO selects the replaced CA and shares a copy of the blocks with the new CA. A publisher/subscriber model between the CA and IoT devices, such as HEMSs, enables HEMSs (subscribers) to receive the CA’s (publisher) latest updates on DR events [44]. HEMS controls household energy resources such as PV panels, EV, and appliance usage.

Blockchain transactions may introduce latency into the system. This delay may not be suitable for real-time demand response scenarios that require rapid decision-making and execution. To mitigate this issue, our system detects the peak load periods (PLPs) and collects the customers’ decisions ahead to reduce the likelihood of time delays. In this proposed architecture, we focus on two events, (1) PLPs events and (2) network supervision events (Sups). The former happens when the demand of the network is more than the DSO threshold, which results in a high cost of supply for the network. Then, at PLP, the DSO sends DR signals for demand reduction to the customers to control the network demand. The latter (Sup) is a supervision event that happens regularly for network demand control, and customers submit their consumption to the CA. The block mining process is required to store the DR transactional data and supervision information on the blockchain. Both $\tau_{PLP}$ and $\tau_{Sup}$ are sets of PLP and network supervision time slots for the next 24 h and we define $h\in \tau_{PLP}$ or $h\in \tau_{Sup}$, where h∈H and H={1,2,…,H} with *H* the number of time slots. The value $N_{Cu}$ is the number of customers in a PB and $N_{Cu}=\left\{1,2,\ldots ,N_{Cu}\right\}$ is the set of customers, where *i* denotes the customer index and $i\in N_{Cu}$.

Note that we use blockchain as a distributed ledger within a smart contract context (see Section 4.2) to validate and authenticate DR transactions, as given by the PoES consensus mechanism (see Section 4.1). The transactions themselves are predicated upon the results of the Stackelberg game, which determines the Nash equilibrium solution regarding customer energy availability and allocated for block mining via financialization by a utility function (see Section 5.1). The utility function employs the consensus value (PoES) as a coefficient when considering the likelihood of winning at block mining.

### 3.2. Authentication Technique

We assume that the DSO and CAs are trusted parties and registered as authenticated nodes in the network. HEMSs require an initial registration to prevent malicious activities and fraudulent information submission on the blockchain. A digital signature algorithm (DSA) can be used for node authentication and for preventing transactional data tampering [45]. In other words, it provides message integrity and node authentication while guaranteeing message security. Consequently, a new customer in the network is obligated to submit initial information such as identity (ID) number, EV total capacity, and PV maximum capacity. In return, the CA generates a public verification key (PK) and a secret/private signing key (SK) for each HEMS using $keygen\left(1^{\lambda }\right)⟶\left(PK,SK\right)$, and λ is a security parameter. Thus, every HEMS has a unique public key and secret/private key pair. A HEMS uses SK to sign a message message while sending it to the network using the algorithm, sign(message, SK)⟶σ. σ is the HEMS signature. The nodes in the network can verify the transaction using the HEMS public key as verify(message, σ,PK)⟶(0, 1), where 1 if the verification is successful and zero otherwise. To reduce the rate of malicious activities, a new HEMS has to provide a security deposit to the CA as a sign of good faith [18,20]. The digital signature authentication technique has been used in our designed smart contract, and it is explained in Section 4.2.

## 4. Blockchain Design

To reveal more details on how the designed energy blockchain works with agents, we summarize our design in four steps. First, a HEMS sends a transaction (including consumption information and digital signature) into the network. Next, the smart contract executes to validate and authenticate the transaction. Then, for the block validation, the consensus algorithm initiates to select a block miner. Finally, the selected miner inserts the validated block into the chain. In summary, we present a new consensus algorithm and a new smart contract design, and use the DSA to guarantee network security and data integrity. Note that in this section, we first explain the consensus algorithm in detail and then talk about the smart contract because customers’ transactions include a consensus parameter that needs to be validated by the smart contract.

### 4.1. Consensus Algorithm—Proof of Energy Saving (PoES)

The required processing hardware for block mining can be satisfied using customer computing hardware for transaction validation and block generation. To implement this, one customer needs to be selected as a block miner. As shown in Figure 1, in a PB, only one HEMS would be a block miner in each round of block generation. This allows the DSO to minimize the consensus energy usage by selecting one miner, unlike the proof of work (PoW) and proof of stake (PoS), and save money on the block generation process. Additionally, the DSO allocates a portion of mining monetary resources to the customers to incentivize them to compete on block mining selection. To this end, our proposed consensus algorithm PoES is presented to efficiently use the energy and hardware resources of customers for block mining.

The mempool in the blockchain is a storing architecture that accumulates the transactions temporarily. After the mempool reaches full capacity, it is time to generate a block and save the data on the blockchain. Our proposed consensus algorithm, PoES, selects one customer with the highest average value of availability, reputation, and compliance ratio as a block miner. The reasons for choosing these factors are threefold: first, to pick a customer with adequate energy (DER resources) to complete the block mining process (availability); second, to select the trusted block miner and prevent malicious activities (reputation); third, to incentivize customers to increase their DR contribution and maximize the chance to win block mining (compliance ratio).

(1) Availability $\left(a_{h}^{i}\in \left[0,1\right]\right)$ presents the ratio of allocated energy for block mining by customer *i* over the required mining energy $\left(\chi_{h}\right)$ at time slot *h*, where we have $a^{i}=\left(a_{1}^{i},\ldots ,a_{H}^{i}\right)$. A customer can supply the block mining energy from its household PV generation, demand (portion of customer desired consumption), and discharge of EV at time slot *h*. The availability is calculated by $a_{h}^{i}=\frac{x_{h}^{i}+w_{h}^{i}+\alpha_{h}^{i}e_{h}^{i}-b_{h}^{i}}{\chi_{h}}$, where $x_{h}^{i}$ is the customer’s consumption from the grid with vector $x^{i}=\left(x_{1}^{i},\ldots ,x_{H}^{i}\right)$, $w_{h}^{i}$ is the PV generation, $e_{h}^{i}$ is the EV discharging energy from customer *i* at time slot *h*, and $b_{h}^{i}$ is the customer minimum-required demand at time slot *h*. In addition, $\alpha_{h}^{i}$ is a binary value that indicates the EV status, where $\alpha_{h}^{i}=1$ means that the EV of customer *i* is parked at home (can be discharged) for time slot *h*, otherwise $\alpha_{h}^{i}=0$. Note that $x_{h}^{i}+w_{h}^{i}+\alpha_{h}^{i}e_{h}^{i}$ would be the customer’s total energy usage at time slot *h*.

(2) Reputation $\left(r_{h}^{i}\in \left[0,1\right]\right)$ customers can rank their experience of interacting with others as trusted, untrusted, or uncertain according to [46]. Then, $r_{h}^{-i}=(r_{h}^{1},\ldots ,\;r_{h}^{i-1},\;r_{h}^{i+1},\ldots ,\;$$r_{h}^{N_{Cu}})$ is the reputation vector customer *i* submits for other customers in the area for time slot *h* before the game starts. The reputation increases and decreases by the number of successful and unsuccessful minings.

(3) Compliance Ratio $\left(c_{h}^{i}\in \left[0,1\right]\right)$ is the ratio of the total electricity reduction of a customer over its total load shed availability (LSA) in a time window, where we have $c^{i}=\left(c_{1}^{i},\ldots ,\;c_{H}^{i}\right)$. We define the compliance ratio of customer *i* for time slot *h* as $c_{h}^{i}=\frac{\delta^{i}+\left(p_{h}^{i}-x_{h}^{i}\right)}{\Sigma l^{i}}$. The value $\delta^{i}$ presents the summation of demand reduction, and $\Sigma l^{i}$ is the summation of the load shed availability of customer *i* in a time window. Finally, $p_{h}^{i}$ is the predicted demand of customer *i* at time slot *h*. Customers can reduce their demand using household PV generation, decrease their actual demand (by reducing or shifting the demand), and discharge EV capacity to handle the request. Hence, a customer with a considerable history of demand reduction (DR contribution) has more chance to be selected as a block miner.

By averaging these three coefficients $arc_{h}^{i}=\frac{a_{h}^{i}+r_{h}^{i}+c_{h}^{i}}{3}$, $\left(arc_{h}^{i}\in {\mathrm{arc}}^{i}\right)$, the consensus value of customer *i* is $arc_{h}^{i}\in \left[0,1\right]$. Finally, the algorithm selects a HEMS with $\max_{\forall i\in N_{Cu}}\left\{arc_{h}^{i}\right\}$ as a block miner for time slot *h*. All the nodes/HEMSs in a private blockchain are aware of each other’s consensus value, and they can easily find the client with the highest consensus value as the block miner of a block mining round. The respective client knows its leader and publishes the block. All other nodes know that this is a valid leader, as they know that the node has the highest consensus value.

### 4.2. Smart Contract

Smart contract is a multistep transaction validation program on blockchain that can prevent disruptive and fake data insertion in a distributed network [47]. After a transaction is submitted, its validity and authenticity need to be verified using the smart contract before being added to the mempool. Every agent located in a PB can execute the smart contract to check the validity of a transaction. However, in this model, only a proportion of HEMSs, who have a reputation value $r_{h}^{i}$ more than the trust threshold $\bar{r}$, are allowed to validate the transaction. Note that the CA decides on the trust threshold $\bar{r}$ and shares the value with all customers. In addition, the trust threshold changes over time based on the local average reputation value. These HEMSs are called active nodes (ANs), and the idea is to reveal the transaction data only with the trusted nodes (active nodes) in the network to keep the privacy of transactions and reduce the overload.

Algorithm 1 shows the steps of our designed smart contract that are executed by active nodes. A transaction contains $a_{h}^{i},\;r_{h}^{i},\;c_{h}^{i}$, and $x_{h}^{i}$ that are signed by HEMS’s private key. First, the transaction authenticity is determined using the HEMS public key to validate the message integrity and authentication. Next, the reputation value is checked $\left(r_{h}^{i}\right)$ to be in a standard range and be equal to the HEMS historical reputation value $rep_{h}^{i}$. Note that the historical reputation value is a copy of the customer *i*’s reputation that is transparent to all HEMSs. The availability value $a_{h}^{i}$ has to be equal to one, otherwise, the corresponding HEMS cannot satisfy the mining energy $\chi_{h}$. In addition, the compliance ratio $c_{h}^{i}$ has to be between 0 and 1, and energy consumption $x_{h}^{i}$ has to be more than zero. Note that the transaction information is only transparent to active nodes. To this end, if a transaction successfully passes these steps (smart contract), it will be added to the mempool.
**Algorithm 1** Smart Contract.1:**Inputs**: $message=(a_{h}^{i},\;r_{h}^{i},\;c_{h}^{i},\;x_{h}^{i})$, PK and σ2:**if** verify(message, σ, PK)⟶1 **then**3:     **if** $a_{h}^{i}==1$ and $r_{h}^{i}==rep_{h}^{i}$ and $0<c_{h}^{i}<1$ and $x_{h}^{i}\geq 0$ **then**4:         The transaction is accepted and added to the mempool.5:   **end if**6:**end if**

It is possible that malicious customers try to deny their submitted energy consumption $\left(x_{h}^{i}\right)$. In this case, our model penalizes customers by seizing their security deposit and reducing their reputation value. Now, we must discover the optimal energy consumption $\left(x_{h}^{i}\right)$ for every customer $\left(i\in N_{Cu}\right)$. Our mixed-strategy Stackelberg game is used to achieve realistic outcomes, as explained in the following section.

## 5. Stochastic Stackelberg Game

### 5.1. Game Model

The DSO regularly monitors the grid and forecasts day-ahead peak load periods by analyzing customer load profiles using the support vector regression (SVR) prediction model. Here, $p_{h}^{i}$ is the customer *i*’s predicted demand for the time slot *h*, and $p^{i}=\left(p_{1}^{i},\ldots ,p_{H}^{i}\right)$ is the vector of customer *i* predicted demand in the next 24 h. As mentioned before, a block generation process is required for both PLP and network supervision events. In the case of network supervision, the DSO asks the CAs to regularly collect customer consumption data from the grid $\left(x_{h}^{i},\;\forall i\in N_{Cu}\right)$. Then, (based on the size of the mempool) the supervision data needs to be stored on the blockchain.

As shown in Figure 1, the DSO passes $\tau_{PLP}$ (in case of DR signal) and $\tau_{Sup}$ (in case of supervision signal) to the CA; then, the CA passes them to the HEMS in its local area. In the case of a DR signal, HEMSs reply to the demand reduction signal with their load shed availability vector $l^{i}=\left(l_{1}^{i},\ldots ,l_{H}^{i}\right)$, where $l_{h}^{i}\in l^{i}$, and then the CA passes them to the DSO. Load shed availability is the desired amount of customer demand reduction for the PLP event. Since the DSO now has a local area demand reduction estimate, it offers the demand reduction reward vector $d=(d_{1},\ldots ,d_{H})$ for $\tau_{PLP}$ and mining reward vector $q=(q_{1},\ldots ,q_{H})$ to the CA. Hence, before the game starts, the customers submit the reputation vector $r_{h}^{-i}$ to the network. At this level, the CA starts the mixed-strategy Stackelberg game and sends the vector of prices k and q to the HEMSs. Note that $k=(k_{1},\ldots ,k_{H})$ is the reward price vector that the CA allocates to customers for demand reduction and we have $d_{h}>k_{h}$, where the difference is considered as the CA profit.

(1) $$\left\{\begin{array}{cc} d_{h},k_{h}\neq 0 & \mathrm{if}\;h\in \tau_{PLP}\\ d_{h},k_{h}=0 & \mathrm{otherwise} \end{array}\right.$$

At the customer level (followers), the customers will receive k (for the PLP event) and q from the CA and maximize their utility function

(2) $$U_{Cu}^{i}=\sum_{h\in Time}\left(q_{h}arc_{h}^{i}+k_{h}\beta_{h}^{i}\right)$$

subject to;

$$x_{h}^{i}+w_{h}^{i}+\alpha_{h}^{i}e_{h}^{i}\geq b_{h}^{i}$$

$$\beta_{h}^{i}=\left\{\begin{array}{cc} 1 & \mathrm{if}\;x_{h}^{i}\leq \left(p_{h}^{i}-l_{h}^{i}\right)\\ \frac{\left(p_{h}^{i}-x_{h}^{i}\right)}{l_{h}^{i}} & \mathrm{otherwise} \end{array}\right.$$

to increase the monetization of demand reduction in the case of PLP and energy resource allocation for block mining. In (2), $x^{i}=\left(x_{1}^{i},\ldots ,x_{H}^{i}\right)$ is customer *i*’s strategy vector. The customer shares $x^{i}$, $a^{i}$, and $c^{i}$ with the CA for every DR and supervision transaction. Note that (2) can be used for both PLP and supervision events according to $Time=\{\tau_{PLP}\;or\;\tau_{Sup}\}$.

In (2), the first part calculates the customer reward of block mining and the second part presents the demand reduction profit. The first constraint keeps the summation of consumption from the grid $\left(x_{h}^{i}\right)$, PV energy production $\left(w_{h}^{i}\right)$, and EV energy discharging $\left(e_{h}^{i}\right)$ more than the customer *i*’s minimum-required energy $b_{h}^{i}$. In addition, $\alpha_{h}^{i}$ indicates the EV status as defined in Section 4.1. Note that the EV can discharge when its capacity at time slot *h* is above a certain threshold $T_{h}$. $\beta_{h}^{i}=1$ when the customer keeps the energy consumption from the grid less than the difference of the predicted demand and LSA $\left(x_{h}^{i}\leq p_{h}^{i}-l_{h}^{i}\right)$; otherwise, if the customer increases the usage $\left(x_{h}^{i}>p_{h}^{i}-l_{h}^{i}\right)$, then $\beta_{h}^{i}=\frac{p_{h}^{i}-x_{h}^{i}}{l_{h}^{i}}$. Note that if the customer increases their consumption more than the predicted demand $\left(x_{h}^{i}>p_{h}^{i}\right)$, they will be penalized with the same utility value $\left(\beta_{h}^{i}=\frac{p_{h}^{i}-x_{h}^{i}}{l_{h}^{i}}\right)$.

At this point, the CA starts the negotiation (the leader–follower game) with customers in the case of demand reduction and tries to keep a proportion of demand reduction rewards ($d_{h}$) for itself. Therefore, it offers k for a PLP event and q for the mining reward to the customers. According to these values, the CA can maximize the utility function

(3) $$\begin{array}{c} U_{CA}=\sum_{h\in \tau_{PLP}}(d_{h}(1-\frac{\sum_{i=1}^{N_{Cu}}x_{h}^{i}}{\sum_{i=1}^{N_{Cu}}p_{h}^{i}})-\sum_{i=1}^{N_{Cu}}k_{h}\beta_{h}^{i}) \end{array}$$

subject to;

$$k_{h}^{min}\leq k_{h}\leq k_{h}^{max}$$

where $k=(k_{1},\ldots ,k_{H})$ is the strategy vector.

Unlike (2), (3) is only used for the PLP event ($h\in \tau_{PLP}$) since $d_{h},k_{h}=0$ for network supervision. Therefore, the game is only played for PLP event; for the supervision event, an optimization is conducted using (2). According to (3), the first part calculates the profit value of the CA which is proportional to the total power consumption of customers $\left(\sum_{i=1}^{N_{Cu}}x_{h}^{i}\right)$ over their predicted demand $\left(\sum_{i=1}^{N_{Cu}}p_{h}^{i}\right)$, and the second part presents the monetary reward that the CA allocates to customers’ demand reduction. The constraint keeps the offered DR reduction price between a lower bound $k_{h}^{min}$ and upper bound $k_{h}^{max}$. The negotiation between CA and HEMSs repeats until both sides reach an equilibrium, where no one has any incentive to change their strategies.

### 5.2. Mixed-Strategy Stackelberg Game and Equilibrium Analysis

The mixed-strategy game model is a stochastic approach that employs a probability distribution model to allocate random values to the strategies of players in different situations [48]. Based on this definition, the mixed-strategy Stackelberg game is used to model the interaction between the CA and customers. Our finite n-player game is defined as follows:

(4) $$\Gamma =(N,\;{\left\{S^{n}\right\}}_{n\in N},\;{\left\{U^{n}\right\}}_{n\in N})$$

where N$\left(N=N_{CA}\cup N_{Cu}\right)$ is a finite set of players and *N* is the total number of players while we have $N_{Cu}=N-1$ customers and $N_{CA}=1$ with the sets of $N_{Cu}$ and $N_{CA}$, respectively. In addition, $S^{n}$ is the space of pure strategies, and $U^{n}$ is the utility function for player n∈N. Γ is a finite game where we have a finite number of pure strategies for players. Therefore, for each customer $i\in N_{Cu}$, $m_{h}^{i}$ is the number of pure strategies at time slot h∈H, and we have $x_{h}^{i}\in S_{h}^{i}=\left\{s_{1}^{i},s_{2}^{i},\ldots ,s_{m_{h}^{i}}^{i}\right\}$. For the CA, we also have $m_{h}^{1}$ number of pure strategies at time slot h∈H and strategy space, $k_{h}\in S_{h}^{1}=\left\{s_{1}^{1},\;s_{2}^{1},\ldots ,\;s_{m_{h}^{1}}^{1}\right\}$.

According to [49], a mixed-strategy equilibrium exists when the players choose their strategies probabilistically. Thus, a mixed-strategy of player *n* at time *h* is presented by a probability distribution vector $\sigma_{h}^{n}=\left(\rho_{1}^{n},\rho_{2}^{n},\ldots ,\rho_{m_{h}^{n}}^{n}\right)$ over $S_{h}^{n}$. The summation of the probabilities of player *n*’s different strategies at time slot *h* is

(5) $$\sum_{d=1}^{m_{h}^{n}}\rho_{d}^{n}=1.$$

For a mixed-strategy game, the probability distribution over the joint pure strategies $s=\left(s_{d^{1}}^{1},\;s_{d^{2}}^{2},\ldots ,\;s_{d^{N}}^{N}\right)\left(s\in S\right),\;$∀n∈N and $d^{n}\in \left\{1,\ldots ,\;m_{h}^{n}\right\}$, is $\sigma \left(s\right)=\left(\rho_{d^{1}}^{1}\;,\rho_{d^{2}}^{2},\ldots ,\;\rho_{d^{N}}^{N}\right)$. Therefore, player *n*’s mixed-strategy payoff value is found as;

(6) $$U^{n}\left(\sigma \right)=\sum_{s\in S}U^{n}\left(s\right)\sigma \left(s\right)$$

$$\sigma \left(s\right)=\prod_{n\in N}\rho_{d^{n}}^{n}$$

where $U^{n}\left(s\right)$ is the utility function of player *n* for joint strategies s∈S and $\sigma \left(s\right)=\prod_{n\in N}\rho_{d^{n}}^{n}$ is its probability distribution. We apply the Monte Carlo model to allocate probabilities to the pure strategies of players based on (5). After calculating (6) for player *n*, the highest payoff value would be selected as the best result, and its related strategy would be the best response.

Several works have proven the existence of a mixed-strategy Stackelberg equilibrium (SE) [50,51,52]. Following these studies, to prove the existence of Stackelberg equilibrium, we first divide the game into mixed-strategy sub-games (a game between HEMSs and CA in PB is considered as a subgame) [53,54], and then based on the Kakutani fixed-point theory [55], the mixed-strategy Nash equilibrium (NE) can be proven for each subgame. Moreover, by using the Simplex algorithm [56], we can prune the searching area and find the optimal points in a short time. A complete step-by-step equilibrium proof is presented in our prior work [57].

To find SE based on (6), the following assumption has to be satisfied for the CA when customers select their best responses:

(7) $$\sum_{d=1}^{m_{h}^{1}}U_{CA}\left(s_{d}^{1},s^{-1^{*}}\right)\rho_{d}^{1}$$

subject to:

$$\sum_{d=1}^{m_{h}^{1}}\left(\sum_{i=1}^{N_{Cu}}\left(U_{Cu}^{i}\left(s_{d}^{1},s_{d^{i}}^{i^{*}}\right)-U_{Cu}^{i}\left(s_{d}^{1},s_{d^{i}}^{i}\right)\right)\right)\rho_{d}^{1}\geq 0$$

$$\sum_{d=1}^{m_{h}^{1}}\rho_{d}^{1}=1$$

$$\forall s_{d}^{1}\in S_{h}^{1}$$

where $s_{d}^{1}$ is the pure strategy of the CA while $s_{d}^{1}\in S_{h}^{1}$. $s^{-1^{*}}$ is a joint variable of customers’ best response strategy when the CA selects strategy $s_{d}^{1}$. The value $\rho_{d}^{1}$ is the probability of the CA, and $s_{d^{i}}^{i^{*}}$ is the pure best strategy of customer *i* where $s_{d^{i}}^{i^{*}}\in S_{h}^{i}$. Equation (7) represents that, for $s^{-1^{*}}$, we calculate a mixed-strategy for the CA via linear programming (LP) and under the constraints, $\left(\rho_{1}^{1^{*}},\ldots ,\rho_{m_{h}^{1}}^{1^{*}}\right)$ would be the optimal mixed-strategy that maximizes the CA utility function.

The equilibrium is found using backward induction that first calculates the best response of customers *i*$\left(s_{d^{i}}^{i^{*}}\right)$ using

(8) $$s_{d^{i}}^{i^{*}}=\underset{A}{\mathrm{argmax}}\sum_{d^{i}=1}^{m_{h}^{i}}U_{Cu}^{i}\left(s_{d}^{1},s_{d^{i}}^{i}\right)\rho_{d^{i}}^{i},\;\forall s_{d}^{1}\in S_{h}^{1}$$

subject to *A* (which represents the constraints in (2)) for all customers, based on the initial price of the CA as $s_{d}^{1}$ strategy.

Then, customers pass their best strategies ($s_{d^{i}}^{i^{*}},\forall i\in N_{Cu}$) to the CA subgame, and the CA can find its best response $s_{d}^{1^{*}}$ using (7). This process repeats between the CA and customers, where customers calculate (8) with the best response of CA in the next rounds.

**Theorem** **1.** *There is a Stackelberg equilibrium in our presented mixed-strategy Stackelberg game based on the optimal solutions of the customers and CA.*

**Proof.**  According to Kakutani fixed-point theory [55], the existence of mixed-strategy NE has to be proven in each subgame (since we divided the Stackelberg game into subgames) to prove the SE for the whole game. Utility function $U_{Cu}^{i}$ is non-empty, non-negative, and continuous, and it is defined on a compact subset of Euclidean space [58]. Furthermore, $U_{CA}^{i}$ is concave, and we can find its maximum point. Moreover, (7) is a linear optimization on the CA’s probability distribution while it is convex and is defined on a compact subset of Euclidean space. Thus, we can assert that there is an optimal point in (7) which is a mixed-strategy Stackelberg equilibrium. □

## 6. Security and Privacy Analysis

This section analyzes the data consistency and security of our proposed model against different attacks. A Sybil attack happens when the attacker defines itself with multiple identities, but this attack easily fails due to the identical public keys [59], where in our model the CA represents public keys for the HEMSs. An appending attack occurs when a registered node in the network tries to submit a fraudulent transaction to the blockchain [19]. To neutralize this attack, our defined smart contract never approves a node with invalid availability, reputation, compliance ratio, and consumption data. Sometimes, a malicious node attempts to alter submitted transaction data or block information; this behavior is referred to as data forgery. [44,47]. In our case, block information cannot be forged due to the hash pointer feature, and the attack could be easily detected with the digital signature. The data spoofing attack changes the identity of HEMSs [44], but in our scheme, the identity of HEMS is known due to its initial registration within the CA. In case of repudiation [44] in our designed model, malicious nodes may try to refuse their submitted energy consumption values. In this situation, our model punishes the customer by seizing their security deposit and reducing their reputation value. An authentication attack occurs when one node tries to forge another node’s identity [44]. Based on our architecture, the private key of HEMS is unique and confidential, which no one knows, thus a malicious HEMS cannot fake another HEMS’s identity. For a man-in-the-middle attack, a destructive node could attempt to change transactions during transmission [60]. However, within the digital signature and smart contract, no faulty or forged data may be added to the block. A denial of service attack happens when a node starts to send multiple transactions [61] to the CA and keeps it busy to take it out of service. In our architecture, only registered nodes are allowed to submit one transaction in response to the CA’s signal. Therefore, additional transactions are ignored. Last but not least is the 51% attack that happens when 51% of customers become attackers and generate fake data for storage on the blockchain [19]. According to [17], the proof of stake (PoS) consensus algorithm confronts the 51% attack within its voting process. Our proposed CA can recognize the fraud settlements at the execution time, and in return seizes the malicious nodes’ security deposit, reduces their reputation, and moves them to a blacklist, where they will not be selected as a miner or active node for a while. To this end, our consensus algorithm (PoES) can prevent the 51% attack with the help of the active nodes on the transaction validation (voting system). The CA decides on the trust threshold $\bar{r}$ and shares the value with all customers. The HEMSs with certain trust thresholds are called active nodes (ANs). Other nodes in the network can reveal the transaction data only with the trusted nodes (active nodes) in the network to keep the privacy of transactions and reduce the overload. Then, in the first step, the malicious nodes can be easily detected by ANs and be penalized.

## 7. Simulation Results

The required energy for the block mining process depends on the number of transactions and the hash function difficulty level of the miner. There are various articles [62,63,64,65] addressing mining energy, where in [65], the authors have found that for a small private blockchain network, a miner with specific hardware (2 core CPU and 8 GB RAM) requires 1 Watt to process one transaction. Consequently, in our proposed architecture with the same hardware features, we need almost χ=3.2 kW (the size of mempool is 3200 transactions). For $N_{Cu}=100$ customers, the CA launches the DR game model by sending 100 initial transactions, and the Stackelberg game repeats for 15 iterations (30 bidirectional transactions) to settle. In the case of network supervision, customers share their consumption with the CA every 15 min. Then, they transmit almost 400 monitoring signals (transactions) in one hour, and according to the mempool size, three block minings are needed to save the 24-h monitoring data on the blockchain.

A dataset of annual consumption for 100 households, provided by Hydro Ottawa Limited, in Ottawa, and a PV energy generation data, provided by the University of Ottawa SUNLAB [66], was selected to evaluate our model. Our analysis focuses on the month of July. To forecast customers’ load profiles for the subsequent 24 h, we employed a SVR model. The proposed architecture was implemented in Python and MATLAB, and we assumed that each household had a PV system (capacity of 4kW), an EV (battery capacity of 40 kWh, maximum discharging power 5 kW), and a HEMS (with 2 cores CPU and 8 GB RAM) for mining and monitoring scheme. To establish a consistent baseline, we set the initial reputation value for all households to 0.1 ($r_{h}^{i}=0.1$, $\forall i\in N_{c}$). We had $d_{h}=0.3\$/$kWh (based on dispatching price) and $q_{h}=3\$/$kWh as the block mining reward.

### 7.1. Effect of EV on Customer Decision

**Figure 2:**
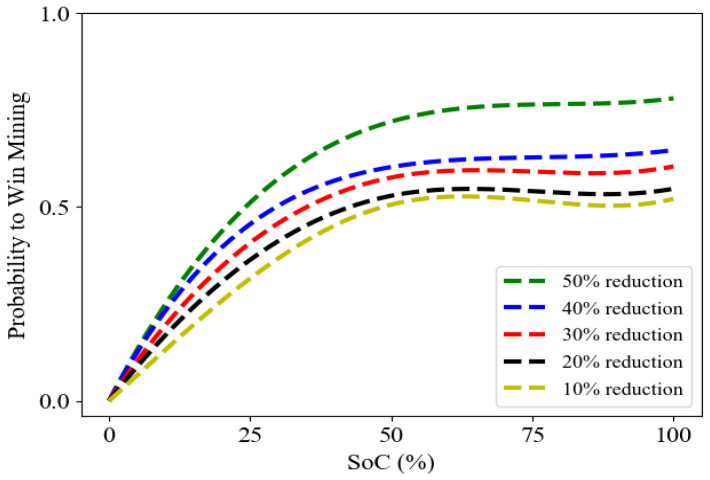
The average probability value of block mining with respect to state of charge (SoC) and different demand reduction percentages.

We made the assumption that every customer has an EV that could be fully charged in 8 h with a maximum discharging power of 5 kW. According to the dataset, the average customer demand would be 1.5 kWh at peak hours. We examined the connection between the probability of winning the block mining and the state of charge (SoC) of the EV battery, while the percentage of demand reduction is fixed. This result is shown in Figure 2. An EV does not discharge its capacity when its SoC falls below $T_{h}=20\%$ (8 kWh). We observed the probability distribution that from zero percent SoC to 35% (14 kWh), the probability mounts with a uniform slope because of the increase in demand reduction, and 35% is an optimal point to start the discharge EV with the maximum power. Thus, for the SoC more than 35%, the probability of winning the block mining raises more than 0.5 for different percentages of demand reduction, which means a higher likelihood of winning the block mining. we found that when demand reduction exceeds 50% of the customer’s baseline load, the probability of winning a block mining increases even further.

**Figure 3:**
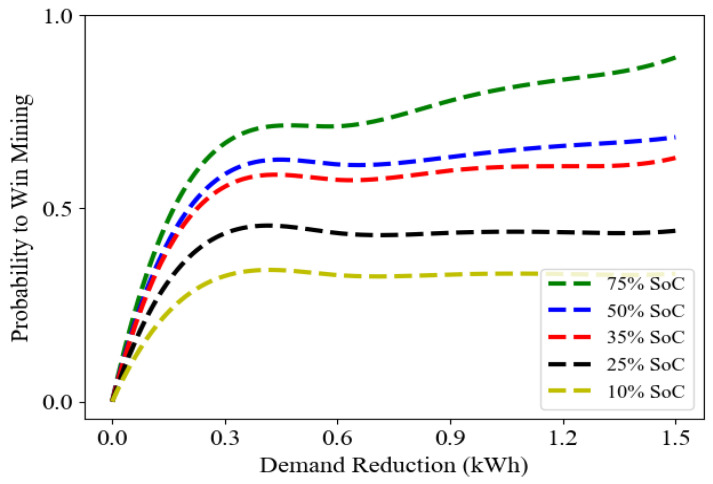
The average probability value of block mining with respect to demand reduction and different state of charge (SoC) percentages.

Next, we examined the impact of various percentages of SoC on the probability of winning the block mining as the demand reduction increased. Figure 3 shows that the average probability increases significantly for low demand reduction, up to about 0.3 kWh, and largely plateauing thereafter, except for the highest SoC, where the probability climbs with increasing DR. That is, not only does the highest SoC win out for a given DR, its likelihood increases for the higher DR at the higher SoC.

**Figure 4:**
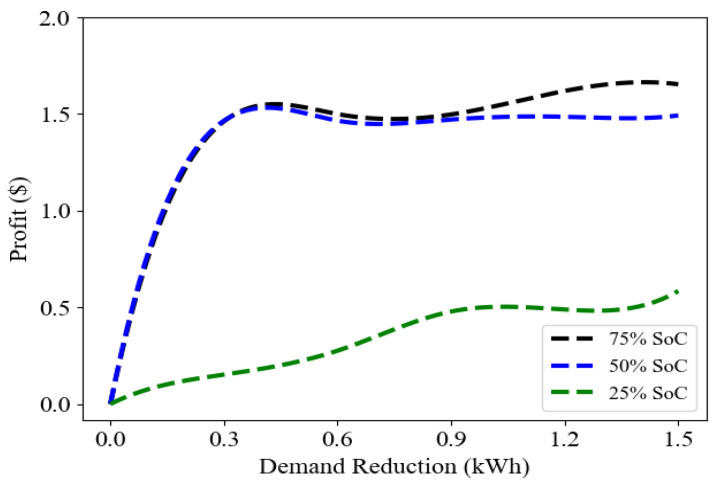
The average profit value of customers with respect to demand reduction and different state of charge (SoC) percentages.

The dependence of the average customer profit, which includes profits from both mining and DR, and the on demand reduction, is presented in Figure 4, with a focus on varying levels of SoC. The customer’s profit is insensitive to SoC at higher values (beyond 50%), increasing strongly for low DR and plateauing at about $1.5/day for higher DR. In contrast, the behavior is far more linear, and quite reduced, for lower SoC, implying that a high SoC is needed to win out at mining.

**Figure 5:**
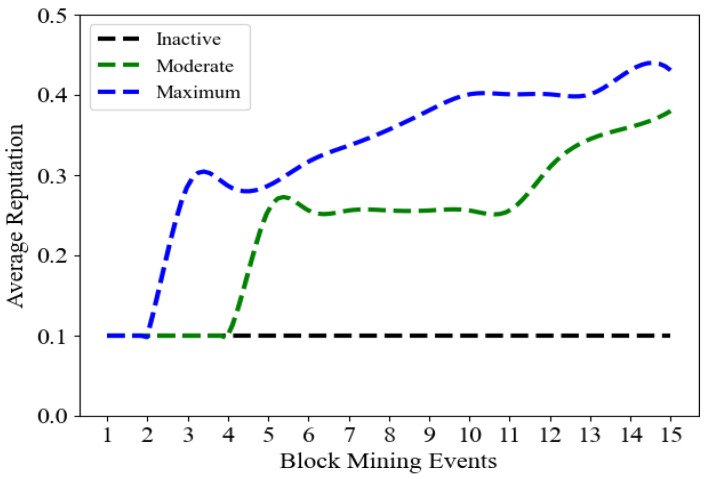
The average reputation value of three different types of customers (inactive, moderate, and maximum) in 15 continuous mining events.

To determine the effect of EV discharging on customers’ average reputation value, Figure 5 displays the reputation changes for three types of customers: inactive (does not discharge EV), moderate (discharge at 50% of discharging rate), and maximum (discharge at the maximum rate) over 15 continuous block mining events. Note that, in this experiment, the demand reduction remained fixed (0.4 kWh) for all customers. The reputation of the maximum customer increases more and it wins more block mining events in comparison. Therefore, we conclude that EVs have significant effects on elevating the chance of customers winning the block mining in addition to increasing their profit by converting their DERs into monetary assets.

### 7.2. Blockchain Performance

**Figure 6:**
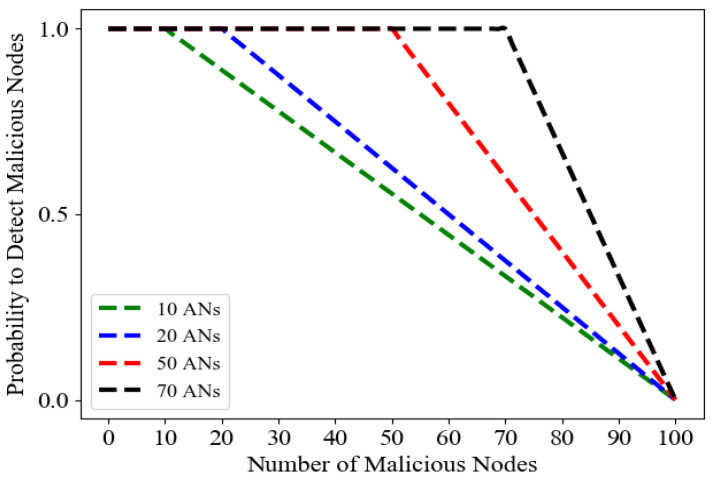
The average probability of detecting malicious nodes with different active nodes (ANs) while the numbers of malicious customers are increasing.

To test the performance of the blockchain in detecting malicious activity, we selected 10, 20, 50, and 70 customers out of a total of 100 customers, as ANs responsible for validating smart contract transactions. The results are shown in Figure 6. We found that with the increase in malicious nodes and the number of ANs in the network, the average probability of detecting the malicious activity is 100% as long as the number of AN is somewhat greater than the number of malicious nodes. Beyond this point, the detection probability decreases linearly with the increasing number of malicious nodes. For example, if half of the customers are malicious and only 10 customers function as active nodes, our model can detect malicious activity with a probability of more than 0.5 and thus defeat a 51% attack. Note that an active node could be a true or malicious node based on a probability distribution model.

**Figure 7:**
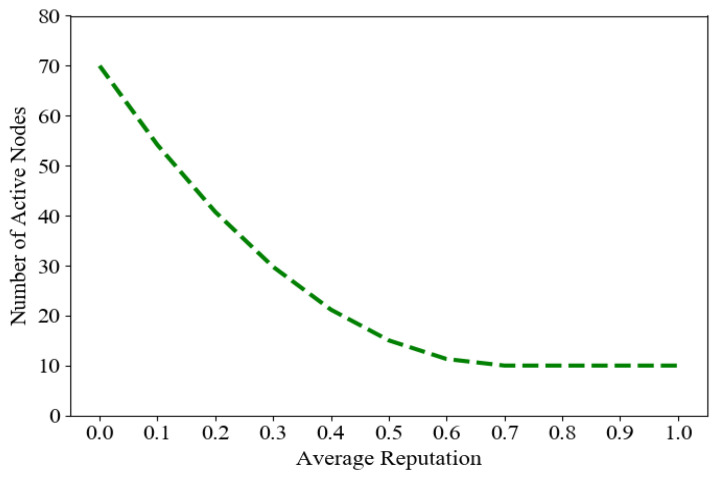
The minimum required number of active nodes (ANs) with respect to the network’s average reputation value.

To determine the minimum number of active nodes needed for secure transaction validation, Figure 7 shows the dependence of the minimum number of ANs needed on the average reputation. We observe that there is a near one-to-one drop in the minimum with increasing reputation, and the minimum needed remains unchanged. We found that, despite variations in the average reputation, the minimum number of active nodes remains constant. It indicates that our proposed blockchain-based mechanisms deliver robustness against malicious activity. Furthermore, the minimum number of active nodes required for secure validation remains relatively stable.

In addition, in Table 1, we performed a comprehensive evaluation of computational costs, communication costs, and storage associated with our proposed mixed-strategy stochastic game, energy transaction validation through smart contracts, and consensus algorithm. The table provides a detailed breakdown of the computational, communication, and storage demands, offering insights into the resource requirements necessary to implement our system. This analysis is required for assessing the feasibility and scalability of our proposed model in real-world settings.

## 8. Conclusions

In this article, we presented a secure and robust DR model that uses the mixed-strategy Stackelberg game integrated with an energy blockchain. The advantages of the proposed solution are: involving customers in a probabilistic game model to control their demand at peak periods, maximizing DR profit for customers, increasing the winning probability for block mining, investing the surplus DERs into monetary resources, and using the distributed processing hardware of the customers for the block mining. We defined the PoES as a consensus algorithm to select the block miner considering the reputation, availability, and compliance ratio. We proved the existence of Stackelberg equilibrium point using Kakutani fixed-point theory. The results presented the relation between the average probability value of block mining and DR contribution and showed that this probability increases by increasing demand reduction and EV’s SoC. We illustrated the average probability of detecting malicious nodes with different ANs, where the proposed energy blockchain model with only 10 active nodes can defeat a 51% attack. We studied the probability of winning the block mining and showed with more than 20% demand reduction and 35% SoC, the probability of block mining goes beyond 0.5. The outcomes showed that the model can provide a secure and robust demand response method by defeating malicious activities and security attacks.

## Figures and Tables

**Table 1:** Cost estimates of components.

Component	Communication	Computational	Storage
Mixed-Strategy Stochastic Game	10 μs	50 μs	0.011 MB
Smart Contract (per validation)	5 μs	500 ns	0.1 MB
Consensus (PoES) (per block validation)	50 μs	0.5 ms	1.5 MB

## Data Availability

Not applicable.

## Abbreviations

The following abbreviations are used in this manuscript:

AP	Access point
AN	Active node
CA	Control agent
DS	Data synchronization
DR	Demand response
DSA	Digital signature algorithm
DER	Distributed energy resource
DSO	Distribution system operator
EV	Electric vehicle
HEMS	Home energy management systems
ID	Identity
IoT	Internet of Things
LP	Linear programming
LSA	Load shed availability
NE	Nash equilibrium
PLP	Peak load period
PV	Photovoltaic
PB	Private blockchain
PoE	Proof of energy
PoEC	Proof of energy consumption
PoEG	Proof of energy generation
PoEM	Proof of energy market
PoES	Proof of energy saving
PoS	Proof of stake
PoW	Proof of work
PK	Public verification key
SK	Secret/private signing key
SaaS	Software as a service
SE	Stackelberg equilibrium
SoC	States of charge
Sup	Supervision event
SVR	Support vector regression
VPP	Virtual power plant
